# Identification of Key Processes that Control Tumor Necrosis Factor Availability in a Tuberculosis Granuloma

**DOI:** 10.1371/journal.pcbi.1000778

**Published:** 2010-05-06

**Authors:** Mohammad Fallahi-Sichani, Matthew A. Schaller, Denise E. Kirschner, Steven L. Kunkel, Jennifer J. Linderman

**Affiliations:** 1 Department of Chemical Engineering, University of Michigan, Ann Arbor, Michigan, United States of America; 2 Department of Pathology, University of Michigan Medical School, Ann Arbor, Michigan, United States of America; 3 Department of Microbiology and Immunology, University of Michigan Medical School, Ann Arbor, Michigan, United States of America; Emory University, United States of America

## Abstract

Tuberculosis (TB) granulomas are organized collections of immune cells comprised of macrophages, lymphocytes and other cells that form in the lung as a result of immune response to *Mycobacterium tuberculosis* (Mtb) infection. Formation and maintenance of granulomas are essential for control of Mtb infection and are regulated in part by a pro-inflammatory cytokine, tumor necrosis factor-α (TNF). To characterize mechanisms that control TNF availability within a TB granuloma, we developed a multi-scale two compartment partial differential equation model that describes a granuloma as a collection of immune cells forming concentric layers and includes TNF/TNF receptor binding and trafficking processes. We used the results of sensitivity analysis as a tool to identify experiments to measure critical model parameters in an artificial experimental model of a TB granuloma induced in the lungs of mice following injection of mycobacterial antigen-coated beads. Using our model, we then demonstrated that the organization of immune cells within a TB granuloma as well as TNF/TNF receptor binding and intracellular trafficking are two important factors that control TNF availability and may spatially coordinate TNF-induced immunological functions within a granuloma. Further, we showed that the neutralization power of TNF-neutralizing drugs depends on their TNF binding characteristics, including TNF binding kinetics, ability to bind to membrane-bound TNF and TNF binding stoichiometry. To further elucidate the role of TNF in the process of granuloma development, our modeling and experimental findings on TNF-associated molecular scale aspects of the granuloma can be incorporated into larger scale models describing the immune response to TB infection. Ultimately, these modeling and experimental results can help identify new strategies for TB disease control/therapy.

## Author Summary

Tuberculosis is a common and deadly infectious disease caused by a highly successful bacterium, *Mycobacterium tuberculosis* (Mtb). Multiple host immune factors control the formation of a self-organizing aggregate of immune cells termed a granuloma in the lungs after inhalation of Mtb. One such factor, tumor necrosis factor-α (TNF), is a protein that regulates inflammatory immune responses. Availability of TNF within a TB granuloma has been proposed to have a critical role in the protective immunity against TB. However, direct measurement of the level of TNF in a granuloma is not experimentally feasible. Therefore, we develop a mathematical model based on an experimental model of granuloma developed in mice to predict TNF availability in a granuloma. We measure values of critical model parameters and explore mechanisms that influence TNF availability in the granuloma. We find that cellular organization in a granuloma and intracellular trafficking of TNF control TNF availability in a granuloma. Further, our model analysis also highlights anti-TNF drug properties that determine their TNF neutralization power. Our findings complement and extend those of recent studies on the role of TNF in the immune response against TB.

## Introduction

Tuberculosis (TB) is caused by a highly successful bacterium, *Mycobacterium tuberculosis* (Mtb), and is responsible for three million deaths per year [1]. 5–10% of infected people fail to control the infection and progress to primary TB disease [2]. A state of latent infection with no clinical symptoms is achieved in most people and may be maintained for the lifetime of the host. However, latent infection can be reactivated years later leading to active tuberculosis. The risk of reactivation is increased in latently infected persons who are elderly, immunocompromised (e.g. due to HIV co-infection), malnourished or taking specific drugs [3], [4]. A key outcome of Mtb infection that arises as a result of the immune response within the host is the formation of aggregates of immune cells and bacteria called granulomas in the lungs. TB granulomas, especially in humans as well as guinea pig and non-human primate models, form as organized spherical structures composed of a core of bacteria, macrophages and dendritic cells (DCs) surrounded by a ring of lymphocytes, including T cells and B cells [2], [5]–[10]. In an infected host with latent infection, the micro-environment created within a granuloma provides appropriate conditions for containment of bacteria [11], [12].

Tumor necrosis factor-α (TNF) is a well-studied inflammatory cytokine that is produced by immune cells, especially activated macrophages and monocytes. TNF is expressed as a 26 kDa membrane-bound precursor protein (membrane-bound TNF; mTNF) that can be cleaved by proteolytic activity of a metalloproteinase TNF-α converting enzyme (TACE) and released as a 17 kDa subunit (soluble TNF; sTNF) into extracellular spaces [13], [14]. Both sTNF and mTNF are trimeric in their mature bioactive form [15] and function by binding to one of the two types of TNF receptors on cells: TNF receptor type 1 (TNFR1; also referred to as p55 or CD120a) and TNF receptor type 2 (TNFR2; also called p75 or CD120b) [16]. Although the two receptors are co-expressed on the surface of most cell types, TNFR1 has been identified as the primary signaling receptor through which most of the inflammatory responses attributed to TNF occur [17]. TNF affects the immune response to Mtb through several mechanisms, including induction of macrophage activation [18], apoptosis [19], [20], and chemokine expression [21]. Further, numerous reports have begun to reveal the role of TNF in granuloma formation as well as in maintenance of granulomas in latent TB [11], [18], [22]–[24]. There are conflicting data, however, regarding the role of TNF in granulomas and Mtb infection and this has arisen because of cross-species comparisons. In humans, anti-inflammatory TNF-neutralizing drugs such as infliximab and etanercept are associated with an increased risk of latent TB reactivation, although the level of susceptibility depends on the drug [25], [26]. Granuloma formation in mice that lack TNF or TNFR1 has been reported to be aberrant or delayed [18]. Neutralization of TNF in mice with chronic infection leads to disorganization of granulomas, increase in bacterial load and subsequent death [23]. However, TNF neutralization in monkeys results in both exacerbation of primary disease and reactivation of latent infection without affecting the granuloma architecture seen in primary and latent TB [27]. Overall, it is clear that TNF plays an important role in TB infection dynamics. Further, TNF availability, i.e. the amount of TNF available to cells in the granuloma, has been reported to be crucial in control of TB infection [28], [29], but there are still open questions regarding the mechanisms controlling TNF availability and the influence of TNF availability on granuloma function.

To elucidate the mechanisms by which availability of TNF in a granuloma is controlled, we focus on TNF interactions with immune cells that comprise a granuloma. We are interested particularly in TNF receptor dynamics. Receptor/ligand interactions at the cell membrane are responsible for initiating intracellular signaling pathways and ultimately the cell response to the external stimulus. However, trafficking events (defined here to include synthesis, internalization, recycling and degradation of ligands and receptors) have been demonstrated to take place under normal physiological conditions and can influence the availability of ligand, the number of ligand-bound receptors and thus receptor-mediated cell responses [30], [31]. TNF/TNFR trafficking processes have been studied in a variety of human and mouse cell lines [32]–[36]. For example, a whole-cell kinetic analysis of TNF/TNFR system with fitting to experimental data on human lung adenocarcinoma A549 cells has shown that the simplest model that reasonably explains the behavior of this system includes receptor synthesis and turn-over, TNF/TNFR association and dissociation as well as TNF/TNFR complex internalization, degradation and recycling of free receptors to the cell membrane [36].

**Figure 1:**
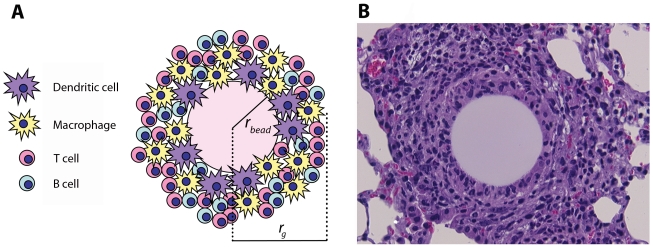
PPD antigen-bead pulmonary granuloma model. (A) Schematic representation (*r_bead_*: radius of bead, *r_g_*: radius of granuloma) and (B) histological appearance of an artificial pulmonary granuloma induced in mouse 4 days after injection of PPD-coated beads [37], [38], [41] (H&E staining; magnification: ×800).

The influence of the dynamics of TNF/TNFR trafficking processes on the availability of TNF in a TB granuloma has never been studied. Thus, we develop a reaction/diffusion-based partial differential equation (PDE) model that describes a TB granuloma as a continuous collection of immune cells forming concentric layers and includes TNF/TNFR binding and trafficking processes. Our multi-scale model is focused on TNF/TNFR-level reactions and interactions, while using a coarse-grain description of the cellular-level details representing a snapshot in time of a granuloma comprised of a static number of immune cells. To analyze the model, we use estimations for TNF/TNFR-associated parameter values from literature and then employ an artificial experimental mouse model of TB granuloma (Figure 1) to quantitatively measure critical model parameters identified by sensitivity analysis. The artificial model of granuloma formation is induced in mice following injection of Sepharose beads covalently coupled to *Mycobacterium* purified protein derivative (PPD) antigen. This model is an appropriate choice for our study as it provides cytokine and cellular patterns that closely match those in an active mycobacterial infection [37]–[41]. Thus our mathematical model also accounts for a bead at the center of the granuloma (Figure 1). We use our model to answer the following questions: What are the most important processes that control TNF availability in a granuloma? Are there likely to be gradients of TNF within a TB granuloma? How does the specific organization of immune cells in the granuloma, i.e. a core of macrophages and DCs surrounded by a mantle of lymphocytes, influence the fraction of TNF-bound receptors and thus TNF signaling for each cell type? And ultimately, how might the neutralization power of TNF-neutralizing drugs in a TB granuloma be affected by their TNF binding properties?

## Methods

### Ethics statement

Animal studies were conducted according to University of Michigan Committee on Use and Care of Animal (UCUCA)-approved protocol (No. 8307).

### TNF/TNFR kinetics at the single-cell level

**Figure 2:**
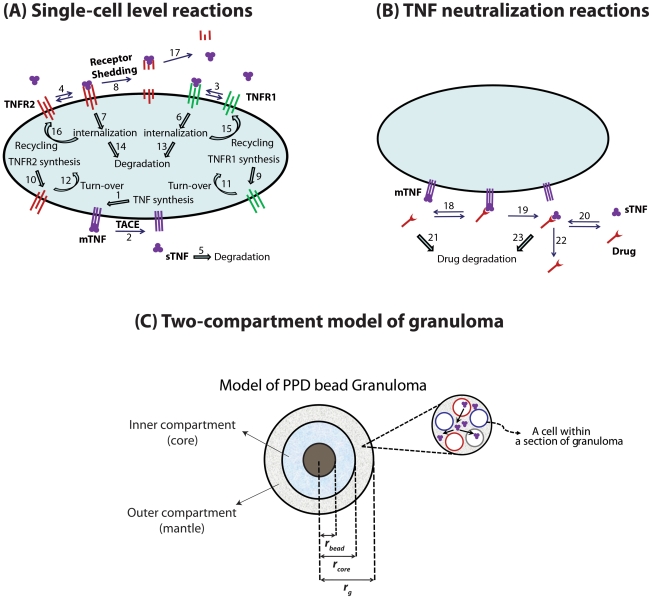
Schematic representation of the multi-scale two-compartment model of PPD bead granuloma and TNF-associated reactions. (A) Binding interactions and reactions controlling TNF/TNFR dynamics at the single-cell level, including synthesis of TNFR1, TNFR2 and mTNF, sTNF release to the extracellular space under the effect of TACE activity, reversible binding of sTNF to TNFR1 and TNFR2, sTNF degradation, internalization of free and sTNF-bound TNFR1 and TNFR2, degradation of internalized TNFR1 and TNFR2, recycling of internalized TNFR1 and TNFR2, shedding of sTNF-bound TNFR2 and release of sTNF from the shed sTNF/TNFR2 complex. (B) TNF neutralization-associated reactions, including reversible binding of drug to mTNF and sTNF, release of drug-bound TNF from the membrane to the extracellular space and drug degradation. (C) Two-compartment model of granuloma that includes a bead of radius *r_bead_* surrounded by the inner compartment populated by macrophages and DCs and the outer compartment composed of lymphocytes. Numbers in (A) and (B) represent model reactions as listed in Table 2.

The binding interactions and reactions controlling TNF/TNFR dynamics at the single-cell level regardless of the cell type are illustrated in Figure 2A. TNF is first synthesized by TNF-producing cells as a membrane-bound precursor form (mTNF) that can then be processed and released as a soluble form (sTNF) into extracellular spaces. This processing occurs via a cell-associated metalloproteinase called TACE [13], [14]. Two types of TNF receptors (TNFR1 and TNFR2) are synthesized and expressed on the cell surface as free receptors. Soluble TNF (sTNF) reversibly binds to TNFRs on the cell membrane or degrades [16], [42], [43]. sTNF-bound cell surface TNFR1 internalizes and sTNF-bound cell surface TNFR2 may undergo internalization or shedding into extracellular spaces [44]. Internalized receptors may degrade or recycle to the cell membrane where they can re-bind to sTNF [36]. Ligand-free TNFRs also turn over (internalize) [34], [36]. Intact sTNF may dissociate from the shed sTNF/TNFR2 complex in the extracellular space [45]. We modeled these molecular processes based on mass action kinetics as shown in Tables 1 and 2; definitions and values of the rate constants are given in Table 3.

**Table 1:** Definition of reaction species.

*Reaction species*			
*mTNF*	Membrane-bound TNF	*sTNF/TNFR1_i_*	Internalized sTNF/TNFR1 complex
*sTNF*	Extracellular soluble TNF	*sTNF/TNFR2_i_*	Internalized sTNF/TNFR2 complex
*TNFR1*	Cell surface TNF receptor 1	*sTNF/TNFR2_shed_*	Shed sTNF/TNFR2 complex
*TNFR2*	Cell surface TNF receptor 2	*Drug*	TNF-neutralizing drug
*sTNF/TNFR1*	sTNF/TNFR1 complex on the membrane	*mTNF/Drug*	mTNF/Drug complex on the membrane
*sTNF/TNFR2*	sTNF/TNFR2 complex on the membrane	*sTNF/Drug*	Extracellular sTNF/Drug complex

**Table 2:** Model reaction and their rates (*v_i_*).

***Base model reactions***		
1	*mTNF synthesis*	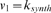
2	*mTNF→sTNF*	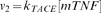
3	*sTNF+TNFR1↔sTNF/TNFR1*	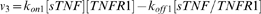
4	*sTNF+TNFR2↔sTNF/TNFR2*	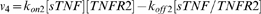
5	*sTNF→degradation*	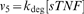
6	*sTNF/TNFR1→sTNF/TNFR1_i_*	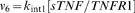
7	*sTNF/TNFR2→sTNF/TNFR2_i_*	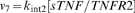
8	*sTNF/TNFR2→sTNF/TNFR2_shed_*	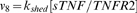
9	*TNFR1 synthesis*	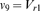
10	*TNFR2 synthesis*	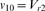
11	*TNFR1→TNFR1_i_*	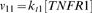
12	*TNFR2→TNFR2_i_*	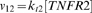
13	*sTNF/TNFR1_i_→degradation*	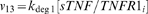
14	*sTNF/TNFR2_i_→degradation*	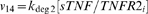
15	*sTNF/TNFR1_i_→TNFR1*	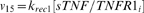
16	*sTNF/TNFR2_i_→TNFR2*	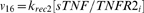
17	*sTNF/TNFR2_shed_→sTNF+TNFR2_shed_*	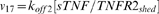
***TNF neutralization reactions***		
18*	*mTNF+Drug↔mTNF/Drug*	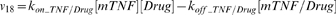
19	*mTNF/Drug→sTNF/Drug*	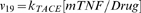
20*	*sTNF+Drug↔sTNF/Drug*	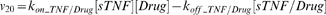
21	*Drug→degradation*	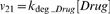
22	*sTNF/Drug→Drug (sTNF degradation)*	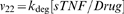
23	*sTNF/Drug→degradation*	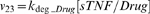

*: Sequential binding of drug to sTNF and mTNF for drugs with TNF binding ratio of greater than 1∶1 was modeled similarly.

**Table 3:** Model parameters, definitions and values estimated from literature.

Parameter	Parameter description	Value*	Reference
*k_synth in_* (#/cell.s)	Average rate of mTNF synthesis in the inner compartment	10^−2^–1	See text
*k_synth out_* (#/cell.s)	Average rate of mTNF synthesis in the outer compartment	0–10^−1^	See text
*R_1 out_* (#/cell)	TNFR1 density in the outer compartment	500–5000	[33], [60], [61]
*R_1 in_* (#/cell)	TNFR1 density in the inner compartment	500–5000	[33], [60], [61]
*R_2 out_* (#/cell)	TNFR2 density in the outer compartment	500–5000	[33], [60],[61]
*R_2 in_* (#/cell)	TNFR2 density in the inner compartment	500–5000	[33], [60], [61]
*f*	Fraction of granuloma in the outer compartment	0.4–0.7	[7], [41]
*D_1_* (cm^2^/s)†	Diffusion coefficient of sTNF	10^−8^–10^−7^ (5.2×10^−8^)	[88], [89]
*D_2_* (cm^2^/s)†	Diffusion coefficient of shed TNF/TNFR2 complex	10^−8^–10^−7^ (3.2×10^−8^)	[88], [89]
*φ* ‡	Volume fraction of the extracellular space per granuloma volume	0.2–0.3 (0.2)	[90], [91]
*d_G_* (#/mm^2^)	Density of granulomas in the lung tissue cross section	0.5–30 (1)	[92], [93]
*ρ* (cell/l)	Mean cell number density in the tissue	6×10^12^	[41]
*r_g_* (µm)	Granuloma radius	100	[41]
*r_m_* (µm)§	Half mean distance between granulomas	1000×(*πd_G_*)^−0.5^	
*r_bead_* (µm)	Bead radius	40	
*r_core_* (µm)	Radius of the inner compartment	[*r_g_* ^3^−*f(r_g_* ^3^−*r_bead_* ^3^)]^1/3^	
*N_av_* (mol^−1^)	Avogadro's number	6.02×10^23^	
*k_TACE_* (s^−1^)	Rate constant for TNF release by TACE activity	10^−4^–10^−3^ (4.4×10^−4^)	[14], [50], [51]
*k_deg_* (s^−1^)	sTNF degradation rate constant	4.58×10^−4^	[74]
*K_d1_* (M)	Equilibrium dissociation constant of sTNF/TNFR1	10^−12^–10^−10^ (1.9×10^−11^)	[33], [42]
*K_d2_* (M)	Equilibrium dissociation constant of sTNF/TNFR2	10^−10^–10^−9^ (4.2×10^−10^)	[33], [42], [53]
*k_on1_* (M^−1^s^−1^)	sTNF/TNFR1 association rate constant	10^7^−10^8^ (2.8×10^7^)	[42]
*k_on2_* (M^−1^s^−1^)	sTNF/TNFR2 association rate constant	10^7^−10^8^ (3.5×10^7^)	[42]
*k_off1_* (s^−1^)	sTNF/TNFR1 dissociation rate constant	*k_on1_*×*K_d1_*	
*k_off2_* (s^−1^)	sTNF/TNFR2 dissociation rate constant	*k_on2_*×*K_d2_*	
*k_int1_* (s^−1^)	TNFR1 internalization rate constant	5×10^−4^–1.5×10^−3^ (7.7×10^−4^)	[42], [44]
*k_int2_* (s^−1^)	TNFR2 internalization rate constant	3.9×10^−4^–5×10^−4^ (4.6×10^−4^)	[53]
*k_shed_* (s^−1^)	TNFR2 shedding rate constant	3.9×10^−4^–1.5×10^−3^ (5×10^−4^)	[44], [50]
*k_rec1_* (s^−1^)	TNFR1 recycling rate constant	8.8×10^−5^–5.5×10^−4^ (1.8×10^−5^)	[34], [36]
*k_rec2_* (s^−1^)	TNFR2 recycling rate constant	8.8×10^−5^–5.5×10^−4^ (1.8×10^−5^)	[34], [36]
*k_t1_* (s^−1^)	TNFR1 turn-over rate constant	3×10^−4^–5×10^−4^ (3.8×10^−4^)	[34], [36]
*k_t2_* (s^−1^)	TNFR2 turn-over rate constant	3×10^−4^–5×10^−4^ (3.8×10^−4^)	[34], [36]
*k_deg1_* (s^−1^)	TNFR1 degradation rate constant	10^−5^–10^−4^ (5×10^−5^)	[32]–[34], [36]
*k_deg2_* (s^−1^)	TNFR2 degradation rate constant	10^−5^–10^−4^ (5×10^−5^)	[32]–[34], [36]
*V_r1 in_* (#/cell.s)	Cell surface TNFR1 synthesis rate constant in the inner compartment	*k_t1_×R_1_in_*	
*V_r1 out_* (#/cell.s)	Cell surface TNFR1 synthesis rate constant in the outer compartment	*k_t1_×R_1_out_*	
*V_r2 in_* (#/cell.s)	Cell surface TNFR2 synthesis rate constant in the inner compartment	*k_t2_×R_2_in_*	
*V_r2 out_* (#/cell.s)	Cell surface TNFR2 synthesis rate constant in the outer compartment	*k_t2_×R_2_out_*	

*: The 25 parameters used for sensitivity analysis are indicated by their ranges of values. Values in parentheses are used to generate other model results.

**†:** Diffusion coefficients of the soluble species in granuloma were estimated in line with estimates for diffusible factors of similar molecular weight in tumors [88], [89].

**‡:** Consistent with extracellular volume fraction estimated for multi –cellular tumor spheroids [90], [91].

**§:** Half mean distance between granulomas were calculated from the granuloma density assessed for 2D sections of the lung tissue [92], [93] and assumed to be consistent in 3D.

### TNF neutralization kinetics

Several TNF-neutralizing drugs have been developed and they work to interfere with TNF activity and thus are used to control inflammation in human diseases such as rheumatoid arthritis and Crohn's disease. These drugs are composed of either monoclonal antibodies (e.g. infliximab) or receptor fusion molecules (e.g. etanercept) that specifically bind TNF, acting as a competitive inhibitor for TNF binding to cell surface TNFRs and eventually neutralizing its functions [46], [47].

To study the effect of TNF-neutralizing drugs of various properties on TNF/TNFR dynamics, we modeled a hypothetical drug as an agent that binds to sTNF or both sTNF and mTNF molecules and also inhibits sTNF binding to both TNFRs. We captured TNF neutralization-associated reactions (schematically shown in Figure 2B) in our model, including reversible binding of drug to mTNF and sTNF [47], [48], release of drug-bound mTNF into extracellular spaces due to TACE activity, and drug or TNF/drug complex degradation [49] based on mass action kinetics as shown in Table 2. Definitions and values of drug-specific parameters are given in Table 4.

**Table 4:** TNF neutralization-associated parameters, definitions, and values.

Parameter	Parameter description	Value	Reference
*D_drug_* (cm^2^/s)*	Diffusion coefficient of drug	2.3×10^−8^	[88], [89]
*k_c_* (cm/s)†	Drug permeability in granuloma	9×10^−7^	[94]
*C_0_* (M)‡	Average drug concentration in the lung tissue	1×10^−7^	[64], [95]
*k_on TNF/Drug_* (M^−1^s^−1^)	TNF/drug association rate constant	10^4^–10^6^	[48], [83], [84]
*k_off TNF/Drug_* (s^−1^)	TNF/drug dissociation rate constant	10^−5^–10^−3^	[48], [83], [84]
*k_deg Drug_* (s^−1^)	Drug degradation rate constant	1×10^−6^	[47]

*: Diffusion coefficient of the drug in granuloma was estimated in line with estimates for diffusible factors of similar molecular weight in tumors [88], [89].

**†:** Drug permeability in granuloma was estimated based on permeability of bifunctional antibodies in tumors [94].

**‡:** Drug concentration in the lung was estimated based on approximate blood concentration of TNF-neutralizing drugs. For most antibodies, tissue/blood concentration ratios are in the range of 0.1–0.5 [95].

### Two-compartment model of granuloma

To study the influence of TNF/TNFR dynamics on the availability of TNF within the multi-cellular structure of the granuloma, TNF/TNFR-associated molecular processes described at the single-cell level were incorporated into a coarse-grain multi-cellular model of a TB granuloma. The model represents a snapshot in time of a granuloma and is composed of an organized collection of a static number of immune cells surrounding a PPD-coated bead. Within this collection, TNF is produced by TNF-producing immune cells, diffuses in extracellular spaces and interacts with TNFR-expressing cells. We modeled the granuloma as a spherical continuum consisting of two cellular compartments. The inner compartment includes evenly distributed macrophages and DCs that form the core of the granuloma, and the outer compartment or mantle is comprised of evenly distributed T cells and B cells (Figure 2C). This is consistent with the structure observed for classical TB granulomas that are comprised of aggregates of macrophages and DCs with a characteristic cuff of lymphocytes, including T cells and B cells on the periphery [2], [6]. Discrete cells are not explicitly considered in this model; each cell-associated species (e.g. cell surface TNF receptor, internalized TNF-bound receptor, etc) is treated as a spatially immobile agent whose concentration in space is expressed by a continuous variable, whereas unbound extracellular sTNF and shed receptors are free to diffuse. Thus, the model includes reaction-diffusion equations for extracellular sTNF and shed receptor concentrations, and basic reaction equations for other species as listed in Table 5. Definitions and values of model parameters are given in Table 3.

**Table 5:** Model equations.

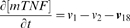
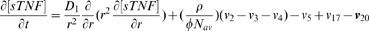
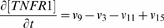
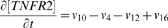
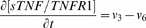
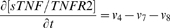
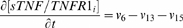
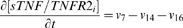
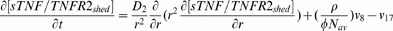
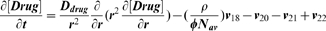
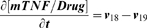
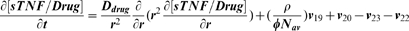

TNF neutralization reactions are distinguished from other reactions by bold font.

To maintain the consistency of the mathematical model with the experimental mouse bead model of granuloma that we study, the granuloma is comprised of a bead of radius *r_bead_* surrounded by cellular layers of the inner and outer compartments with radii of *r_core_* and *r_g_*, respectively (Figure 2C). We assumed no flux of sTNF at *r* = *r_bead_* and at *r* = *r_m_*, a distance equal to half the mean distance between granulomas, due to symmetry with tissue surrounding adjacent granulomas. Initial conditions for TNFRs are specified as:
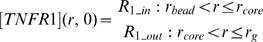
(1)

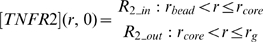
(2)where *R_1_in_*, *R_1_out_*, *R_2_in_* and *R_2_out_* are the average TNFR1 and TNFR2 densities on the membrane of cells in the inner and outer compartments. These parameters were set equal to the steady state concentrations of cell surface TNFRs in each compartment in the absence of TNF and are controlled by the rates of receptor synthesis and turnover of free receptors as indicated in Table 3. Similarly, we assumed the steady state concentration of mTNF (found from Equation 3) in each compartment as the initial value of mTNF for that compartment (Equation 4).
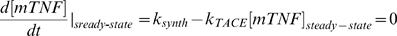
(3)

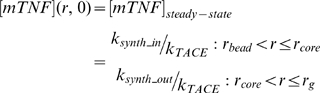
(4)Initial concentrations of other species were set to zero, as cell-associated mTNF is the initial source of the whole TNF in the granuloma.

The PDE model was solved numerically using COMSOL Multiphysics 3.4 (COMSOL AB, Stockholm, Sweden) with MATLAB 7.5 (The MathWorks, Natick, MA). Simulations were run until a steady state was reached (approximately 12 hours of real time). Because TNF-associated molecular level processes studied here occur significantly faster than cellular level events that may change the structure of a granuloma (e.g. cell recruitment, migration and death), we assumed that the structure of granuloma is not changed during the 12-hour time course of simulations.

### Distinct cell types and cellular organization in the granuloma model

To study the influence that specific cellular organizations may have on the availability of TNF within a TB granuloma, we explicitly incorporated major granuloma-comprising cell types (determined from experiments performed herein), including macrophages, DCs, T cells and B cells into our mathematical model of a granuloma. We defined a metric, *separation index* (*s*), to present the level of separation between different cell types in a granuloma, defined as:
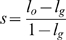
(5)where *l_o_*, *l_g_* are the lymphocyte (T cells and B cells) fractions in the outer compartment and in the whole granuloma, respectively. Thus a separation index of 0 is equivalent to a totally mixed cellular organization, whereas a separation index of 1 represents a separate cellular organization approximately as observed in human and non-human primate models of TB in which DCs and macrophages reside in the inner compartment (core) and lymphocytes compose the outer compartment (mantle). A schematic representation of the effect of changing *s* on cellular organization of the bead granuloma is shown in Supplementary Figure S1(A). Some model parameters were also defined or modified based on consideration of the cellular organization in the model as shown in Supplementary Table S1. For example, using the mean cell volume in each compartment, cell number densities in the inner and outer compartments (*ρ_in_* and *ρ_out_*) are computed and replace general cell density *ρ* in diffusion equations of Table 5.

We assumed that some TNF/TNFR kinetic parameters, including the rate constants for TNF release by TACE activity, TNF/TNFR association and dissociation as well as TNFR internalization, shedding, degradation and recycling have the same values for different cell types. This assumption is based on consistency of experimental data on measurement or estimation of values of these parameters for a variety of cell types including various cell lines expressing TNF and/or TNF receptors with one another as well as other data on similar mammalian cell surface receptors [14], [30], [32]–[34], [36], [42], [44], [50]–[53]. However, the rate of synthesis of TNF and TNFRs depends on the cell type (see Results). Thus, when different cell types are considered, average rates of mTNF synthesis in granuloma compartments can be computed as follows:
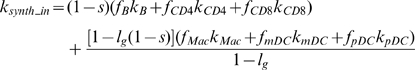
(6)

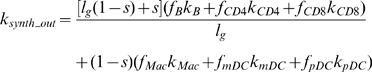
(7)where definitions of parameters are given in Supplementary Table S1. Similarly, average values of TNFR1 and TNFR2 densities in each compartment can be computed.

### Model outputs

The protective role of TNF in immunity to Mtb infection has been shown to depend primarily on the soluble form (sTNF) and its interactions with TNFR1 [18], [54], suggesting that the spatial profile of sTNF concentration and the fraction of sTNF-bound cell surface TNFR1 are model outputs of interest. Therefore, we introduce four steady-state spatially averaged metrics to characterize our simulation results for availability of TNF in a granuloma. These metrics were used to perform sensitivity analysis and include: sTNF-bound fraction of cell surface TNFR1 in the whole granuloma (output 1), granuloma core (inner compartment; output 2) and mantle (outer compartment; output 3) as well as free sTNF concentration in the whole granuloma (output 4).

### Sensitivity analysis

To identify parameters that significantly influence the outcomes of the two-compartment model of a granuloma, we used Latin hypercube sampling (LHS) [55]–[59] to sample values of 25 parameters from the ranges (with uniform distributions) listed in Table 3. Ranges of TNF/TNFR affinity and kinetic parameter values were obtained from a variety of literature data from different cell lines. However, no experimental values are available for several other parameters, including the rate of mTNF synthesis and TNFR densities as well as cell fractions and densities in a granuloma. Thus, relevant ranges of values of these parameters, though not derived from TB granulomas, were used for sensitivity analysis. For example, reported rates of TNF synthesis by activated cultured immune cells [28], [38], receptor densities on human monocytes and lymphocytes [33], [60], [61], and immune cell fractions in the lungs of Mtb infected or mycobacterial antigen activated mice [7], [41] were used.

To reduce the number of parameters in LHS simulations, we replaced distinct cell type fractions with a general parameter *f* defined as the fraction of granuloma in the outer compartment. The parameter *f* directly determines the thickness of the inner and outer compartments as indicated in Supplementary Figure S1(B). The rate of mTNF synthesis and density of TNF receptors were sampled independently as average values of these parameters in each compartment (*k_synth_in_*, *k_synth_out_*, *R_1_in_*, *R_1_out_*, *R_2_in_*, *R_2_out_*). Thus, these parameters together with *f* determine the overall rate of TNF and TNFR expression in the granuloma. Note that the separation index (*s*) defined above is not used in the absence of distinct cell types (i.e. for the sensitivity analysis described here). Simulations sampled each parameter 1000 times, producing 1000 solutions to the model equations. To determine the correlation between parameter values and each of the model outputs, partial rank correlation coefficient (PRCC) values were calculated [55], [56], [62]. PRCC values vary between −1 (perfect negative correlation) and 1 (perfect positive correlation) and can be differentiated based on *p*-values derived from Student's *t* test. The choice of number of simulations (*N*) is determined by the desired significance level for the PRCC [55], [63] and here *N* = 1000 implies that PRCC values above +0.09 or below −0.09 are significantly different from zero (*p*<0.001). Model parameters then were categorized for their significance in affecting the model outputs based on their PRCC values.

### Simulation of TNF neutralization in granuloma

To study the effect of TNF-neutralizing drugs of various properties on TNF availability in a granuloma, the model was run in the absence of drug until a steady state was reached and then the drug was added. We modeled the drug source as a concentration *C_0_* in the surrounding tissue with a flux into a granuloma that is dependent on drug permeability *k_c_* and the drug gradient at granuloma radius *r* = *r_g_*:
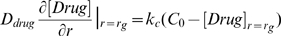
(8)
*C_0_* was considered constant within the time course of simulation that is significantly shorter than the decay time reported for TNF-neutralizing drugs [47], [64]. Equations describing drug/TNF interactions and reactions are listed in Table 5. Drug-associated model parameters are listed in Table 4. To compare the influence of drugs with different properties (parameters) on availability of TNF in a granuloma, TNF neutralization efficiency, *E*, was defined as a function of the ratio of the spatially averaged steady-state concentration of sTNF before drug addition to the spatially averaged concentration of sTNF when drug exerts its steady state maximum effect, i.e. approximately 6 hours after drug addition.
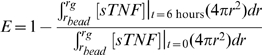
(9)where *t* = 0 stands for the steady state condition at which drug was added.

### Mouse model of TB granuloma

We use an artificial mouse model of TB granuloma that has been demonstrated to provide a well-circumscribed lung granuloma typified by a type 1 cytokine phenotype characterized in TB [38]. Briefly, granulomas were induced in pre-sensitized CBA/J mice lungs following i.v. injection of 6000 Sepharose 4B beads (in 0.5 ml of PBS) covalently coupled to *Mycobacterium* purified protein derivative (PPD) as previously described [38],[40],[41],[65]. After 2 days, PPD-coated beads are surrounded by immune cells including macrophages, DCs, T cells and B cells. PPD-bead granulomas achieve their maximal size on day 4 and gradually diminish thereafter [38]. To measure parameters of interest in PPD bead granulomas, groups of mice were sacrificed at 2 and 4 days after bead injection. Intact granulomas were isolated following homogenization of lungs in cold RPMI-1640 medium (BioWhittaker) in a Waring blender with a narrow-bottom stainless steel cup. Granuloma cells were obtained following a 30-minute treatment of isolated granulomas in a solution of RPMI supplemented with 10% fetal calf serum (FCS), 1 mg/ml collagenase A (Roche) and 30 µg/ml bovine pancreatic DNase I (Sigma) at 37°C and used for further experiments.

### Cellular composition of PPD bead granulomas

To identify the cellular composition of PPD bead granulomas, we used multi-color flow cytometry with fluorescing antibodies specific for immune cell markers, including macrophages, DCs, T cells and B cells. Other immune cells such as neutrophils and eosinophils were not quantified as they have been shown to constitute only a tiny fraction of PPD bead granulomas [41]. The following antibodies/conjugates were used for staining of the cells: anti-CD11b-APC (BD Pharmingen), anti-CD11c-FITC (BD Pharmingen), anti-F4/80-APC-Cy7 (eBioscience), anti-B220-PerCP-Cy5.5 (BioLegend), anti-CD4-PE-Cy7 (BD Pharmingen) and anti-CD8a-Biotin (BD Pharmingen)/Streptavidin-Pacific Orange (Invitrogen). Dead cells were identified and excluded from analysis by staining with the Live/Dead Fixable Violet Dead Cell Stain Kit (Invitrogen). 2×10^5^ events were counted using a BD-LSRII system flow cytometer (BD Biosciences). F4/80^+^ CD11b^+^ macrophage, B220^+^ CD11c^+^ lymphoid dendritic cell (pDC), CD11b^+^ CD11c^+^ myeloid dendritic cell (mDC), B220^+^ B cell, CD4^+^ T cell and CD8^+^ T cell populations were gated following compensation for fluorochrome spectral overlaps. Cell fractions in granulomas were identified following analysis by FlowJo software (Treestar, Ashland, OR).

### TNF receptor quantification

To quantify the number of TNFR1 and TNFR2 molecules on the membrane of each cell type, we used quantitative flow cytometry with Phycoerythrin (PE)-conjugated anti-TNFR1 or -TNFR2 antibodies (BioLegend), together with staining of cell-specific markers as described above. Thus, cell suspensions at a concentration of 1×10^6^ cells per volume of 200 µl were stained with saturating concentrations of antibodies that were identified to be 2 mg/l for anti-TNFR1 and 1 mg/l for anti-TNFR2 antibodies. We generated a calibration plot from the fluorescence intensity measurements on Quanti-BRITE PE-conjugated standard micro-beads (BD Biosciences). This plot was used (after compensation for fluorochrome spectral overlaps) to quantify TNFR1 and TNFR2 densities on the membrane of granuloma macrophages, mDCs, pDCs, B cells, CD4 and CD8 T cells based on the PE mean fluorescence intensities.

### Quantification of the rate of TNF synthesis

Because TNF is initially synthesized as a membrane-bound molecule (mTNF), we can also use quantitative flow cytometry to quantify the rate of TNF synthesis by granuloma-comprising cells, including macrophages, DCs, B cells and T cells. Live granuloma cells were first isolated (from the lungs of a group of 10 mice) by using a Dead Cell Removal Kit (Miltenyi Biotec) and incubated at 37°C in a 5% CO_2_ humidified environment for 4–5 hours. TNF-α proteinase inhibitor-1 (TAPI-1; Calbiochem) at a concentration of 100 µM was added to inhibit TNF release from cell membranes [50], [66]. PE-conjugated anti-TNF antibody (BioLegend) was then used to stain 1×10^6^ cells at a sequence of time-points within a 3 hour period of TACE inhibition at 37°C.

The rate of mTNF synthesis for distinct TNF-producing cell types was derived by nonlinear regression of the experimental data to an equation of the form *y* = *ae^bt^* + *c* as the general form of Equation 10 (which is derived from Equation 3) using MATLAB. Parameters *a*, *b*, *c* then were used to calculate *k'_TACE_* and *k_synth_*.
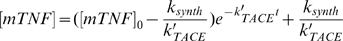
(10)where [*mTNF*]_0_ is the steady-state initial number of mTNF on the cell membrane and *k'_TACE_* is the TNF release rate constant in the presence of TAPI-1 (*k'_TACE_*≤*k_TACE_*). Knowing *k_synth_* and the steady-state initial number of mTNF [*mTNF*]_0_, the value of *k_TACE_* can be calculated from:
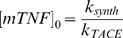
(11)


## Results

### TNF availability within a granuloma

**Figure 3:**
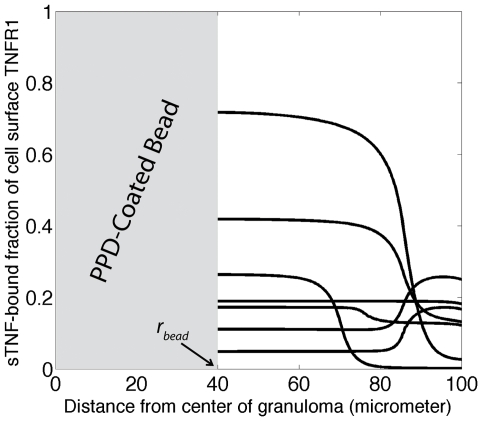
Simulation results for the steady-state profile of sTNF-bound fraction of cell surface TNFR1 in a granuloma using seven different sample sets of parameter values within ranges specified in Table 3. Arrow indicates radius of the bead (*r_bead_*). Parameter values for the particular curves shown are listed in Supplementary Table S2.

To understand the extent to which granuloma properties (e.g. cellular composition, TNFR expression and the rate constant for receptor internalization) impact the availability of TNF, simulations were run for different values of model parameters within ranges given in Table 3. Although TNF exists in different forms in the granuloma, the amount of TNF associated with TNF receptors, and in particular TNFR1, has been identified as a primary factor that determines the outcomes of TNF signaling in the granuloma [18], [67]. Thus, we present steady state model predictions for spatial profile of the fraction of sTNF-bound TNFR1 in a granuloma using several different sample sets of values for model parameters within ranges specified in Table 3 (Figure 3). Simulation results for the spatial profiles of other forms of TNF in the model (soluble and cell-associated sTNF-bound TNFR2 and internalized sTNF-bound TNFRs) are presented in Supplementary Text S1 and Figure S2. Our modeling results demonstrate that TNF availability in granuloma compartments is dramatically influenced by the values of model parameters, including rate constants for TNF/TNFR trafficking events, TNFR densities and the rate of TNF synthesis in granuloma compartments. However, modeling results here are limited in their applicability due to parameter uncertainty, especially uncertainty in the level of TNF and TNFR expression by distinct granuloma-comprising cells. Therefore, we next turn to the identification of critical model parameters that influence the outcome of the model, TNF availability and binding to TNFRs in the granuloma.

### Sensitivity analysis: identifying critical model parameters that influence TNF availability

To identify parameters that significantly influence the availability of TNF within a granuloma, sensitivity of the outputs of the model describing TNF trafficking in a granuloma to changes of input parameters was explored. Table 6 indicates significant PRCC values for model parameters and outputs. For example, the average sTNF-bound fraction of cell surface TNFR1 in the whole granuloma (output 1) was shown to be significantly influenced by a variety of parameters, including the average rate of mTNF synthesis in the inner and outer compartments (*k_synth_in_* and *k_synth_out_*), the average TNFR1 density in the inner and outer compartments (*R_1_in_*, *R_1_out_*), the outer compartment fraction of granuloma (*f*) as well as both TNF receptor affinities for sTNF (*K_d1_* and *K_d2_*) and the rate constant for TNF-induced internalization of TNFR1 (*k_int1_*). Indeed, using different values of these particular parameters as inputs leads to outputs similar to those shown in Figure 3 (data not shown). Thus, experimentally determined values of these parameters are required for generation of useful model predictions.

**Table 6:** Significant PRCC values for model parameters and four spatially averaged steady-state outputs: (1) sTNF-bound fraction of cell surface TNFR1 in the whole granuloma, (2) sTNF-bound fraction of cell surface TNFR1 in the inner compartment, (3) sTNF-bound fraction of cell surface TNFR1 in the outer compartment, (4) sTNF concentration in the whole granuloma.

Parameter	Parameter description	Output (1)	Output (2)	Output (3)	Output (4)
***k_synth_in_***	Average rate of mTNF synthesis in the inner compartment	0.93**	0.93**	0.71**	0.88**
***k_synth_out_***	Average rate of mTNF synthesis in the outer compartment	0.31**		0.82**	0.29**
***R_1_out_***	TNFR1 density in the outer compartment	−0.76**		−0.85**	−0.29**
***R_1_in_***	TNFR1 density in the inner compartment	−0.62**	−0.86**	−0.54**	−0.76**
***R_2_out_***	TNFR2 density in the outer compartment			−0.17**	
***R_2_in_***	TNFR2 density in the inner compartment	−0.09*	−0.15**	0.25**	
***f***	Fraction of granuloma in the outer compartment	−0.49**		−0.32**	−0.36**
***D_1_***	Diffusion coefficient of sTNF			0.19**	
***D_2_***	Diffusion coefficient of shed TNF/TNFR2 complex			0.08*	
***φ***	Volume fraction of the extracellular space per granuloma volume				
***d_G_***	Density of granulomas in the lung tissue				
***k_TACE_***	Rate constant for TNF release by TACE activity				
***K_d1_***	Equilibrium dissociation constant of sTNF/TNFR1	−0.12**	−0.18**	0.16**	0.72**
***K_d2_***	Equilibrium dissociation constant of sTNF/TNFR2	0.14**	0.18**		0.09*
***k_on1_***	sTNF/TNFR1 association rate constant				−0.47**
***k_on2_***	sTNF/TNFR2 association rate constant				
***k_int1_***	TNFR1 internalization rate constant	−0.76**	−0.72**	−0.75**	−0.42**
***k_int2_***	TNFR2 internalization rate constant				
***k_shed_***	TNFR2 shedding rate constant				
***k_rec1_***	TNFR1 recycling rate constant				−0.09*
***k_rec2_***	TNFR2 recycling rate constant				0.09*
***k_t1_***	TNFR1 turn-over rate constant				
***k_t2_***	TNFR2 turn-over rate constant				
***k_deg1_***	TNFR1 degradation rate constant				
***k_deg2_***	TNFR2 degradation rate constant				

Non-significant PRCC values are not indicated.

*: 0.001<p-value<0.05.

**: p-value<0.001.

Parameters that positively correlate with the sTNF-bound fraction of cell surface TNFR1 in the whole granuloma (output 1 in Table 6) include the rate of mTNF synthesis in both compartments and the equilibrium dissociation constant of TNFR2 (*K_d2_*) as a competitor of TNFR1 for binding to sTNF. Conversely, TNFR1 internalization rate constant *k_int1_*, TNFR1 density in both compartments, equilibrium dissociation constant of TNFR1 (*K_d1_*), and the outer compartment fraction of granuloma *f* negatively correlate with this same output. Although greater affinity of TNFR1 for sTNF enhances the level of sTNF binding to TNFR1 in the core of granuloma (output 2) as the major TNF-producing compartment, it reduces the access of TNFR1 on the membrane of cells in the outer compartment to diffusing sTNF (output 3). Thus, increasing the effective diffusion coefficient of sTNF in the granuloma increases the sTNF-bound fraction of receptors in the outer compartment. Diffusion of shed sTNF-bound TNFR2 complex from the inner compartment to the outer compartment of granuloma can also explain the positive correlation of TNFR2 density in the core with the sTNF-bound fraction of TNFR1 in the outer compartment (output 3), while it is negatively correlated with the same response in the inner compartment (output 2) due to competition between receptors for binding to sTNF. Significant correlations of model parameters with the level of free sTNF concentration in the granuloma (output 4) are qualitatively similar to their correlations with output 1, except for *K_d1_* and sTNF/TNFR1 association rate constant *k_on1_* that are, respectively, positively and negatively correlated with output 4.

Applicability of the model will require that we have accurate values of the significant parameters found via sensitivity analysis or else we will have to consider the wide range of possibilities hinted at in Figure 3. There are two different classes of these significant parameters. One class includes parameters associated with TNF/TNFR interactions and intracellular trafficking. The parameters of this class have been theoretically estimated or experimentally measured in multiple cell lines expressing TNF receptors. These studies show that the time scales and thus the rates of significant TNF/TNFR-associated processes identified above are consistent over different cell lines. For example, the obtained TNF/TNFR association and dissociation rate constants for TNFR1 and TNFR2 on a variety of cell lines, including U937, HeLa, and KYM-1 cells were found to be similar and consistent with the data on mouse embryonic fibroblasts [42], [52]. Further, internalization of the sTNF/TNFR1 complex has been shown to occur with a half-time of 10–20 minutes which gives an average value of 7.7×10^−4^ s^−1^ for the TNFR1 internalization rate constant [42], [44]. The values of these parameters are given in Table 3 (in parentheses). The second class of significant parameters are the ones for which no experimental values are available and include cellular fractions, the rate of mTNF synthesis and TNFR densities on immune cells in a TB granuloma. Thus, we measure the values of these parameters in an experimental model of TB granuloma.

### Cellular composition of PPD bead granulomas

**Figure 4:**
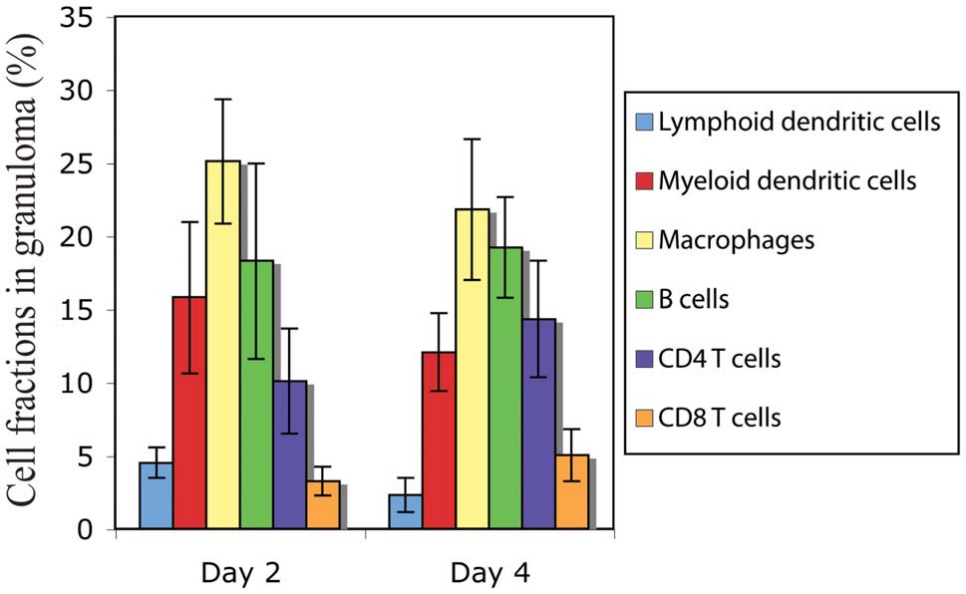
Cellular fractions in PPD bead granulomas at 2 and 4 days of granuloma formation in thirty CBA/J mice quantified by multi-color flow cytometry. Results are expressed as the percentage of each cell type in the total population of granuloma cells. Error bars represent standard deviation from the mean.

We used an artificial model of TB granuloma developed in mice following injection of PPD-coated beads to measure model parameters of interest. To identify the cellular composition of PPD bead granulomas, multi-color flow cytometry with fluorescing antibodies for specific immune cell surface markers was used as described in Methods. Figure 4 indicates experimental data on fractions of the major granuloma-comprising immune cells, including DCs, macrophages, T cells and B cells, that compose approximately 80% of the total cell population of day 2 and day 4 granulomas. Macrophages and B cells were observed to be the largest cell populations in isolated granulomas. A small but statistically significant increase (*p*<0.001) in the percentage of both CD4 and CD8 T cells which represent the adaptive immune response was observed in day 4 granulomas compared with day 2 granulomas. On the other hand, macrophages and DCs were shown to form a slightly smaller portion of day 4 granuloma cell population. The percentage of B cells in granulomas did not significantly change from day 2 to day 4. Cellular composition of the granuloma and the increase in the level of T cell recruitment with time are qualitatively consistent with the experimental data on the infiltration of immune cells into the lungs of mice infected with Mtb as well as data on granulomas induced in lungs of Mtb-infected monkeys, although T cell recruitment occurs in a shorter time scale for PPD bead granulomas [7], [9].

### Quantification of TNFR densities

The average numbers of TNFR1 and TNFR2 molecules on the membrane of day 2 and day 4 granuloma-comprising immune cells were quantified by flow cytometry using standard PE-conjugated beads as described in Methods. DCs, macrophages and B cells were found to be the major TNFR-expressing cells in granulomas with average TNFR1 density of the order of 10^3^ molecules per cell and a lower level of expression for TNFR2 (Table 7). Further, except for lymphoid DCs and B cells that show a significant decrease with time, the level of TNFR expression was similar for day 2 and day 4 granuloma cells.

**Table 7:** Average numbers of TNF receptors per cell quantified by multi-color flow cytometry for different types of granuloma-comprising immune cells isolated from 15 mice.

Cell type	Number of receptors at day 2		Number of receptors at day 4	
	TNFR1	TNFR2	TNFR1	TNFR2
Lymphoid dendritic cells	4600±1100	1900±600	1700±500	1700±1100
Myeloid dendritic cells	1500±400	500±200	1700±400	700±300
Macrophage	1000±300	400±200*	1000±300	500±200
B cells	1100±600	900±200	500±200	200±100*
CD4 T cells	300±100*	400±100*	200±100*	200±100*
CD8 T cells	300±100*	200±100*	100±100*	200±100*

*: PE fluorescence intensity was smaller than the fluorescence intensity of the QuantiBRITE standard beads with the smallest number of conjugated PE molecules.

### Quantification of the rate of mTNF synthesis

**Figure 5:**
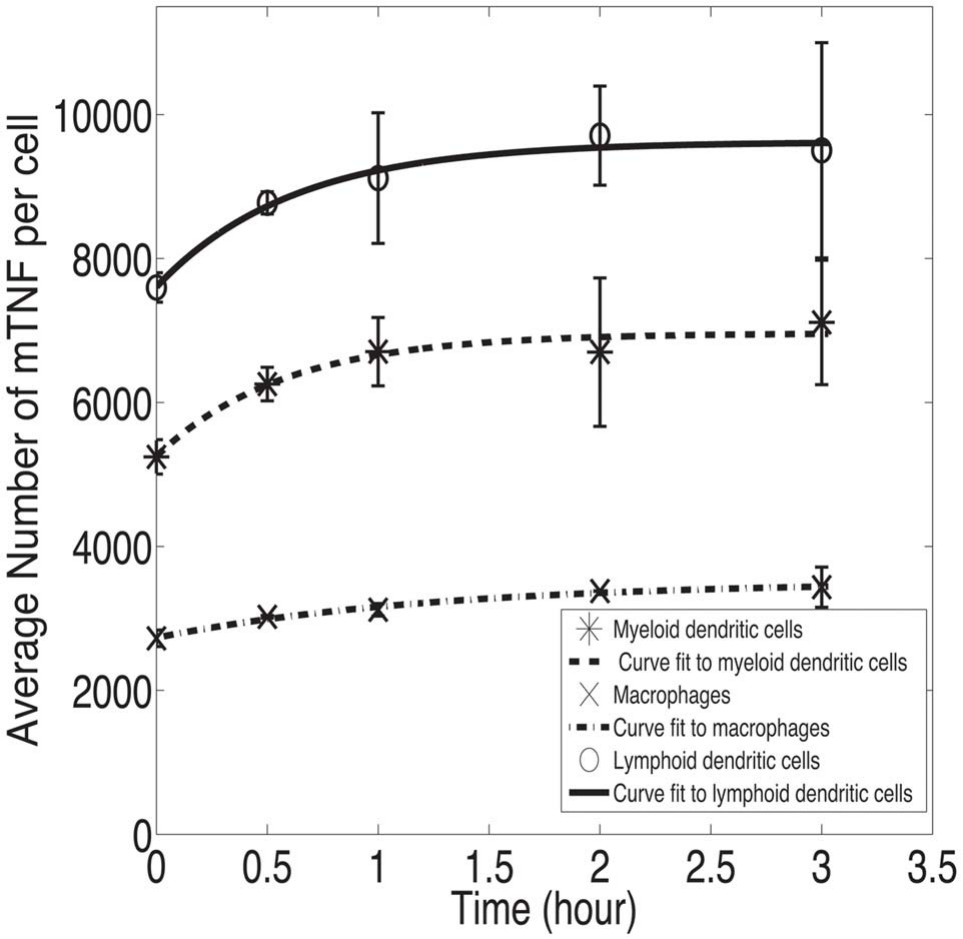
Quantification of the rate of mTNF synthesis by each cell type. Experimental data on the number of mTNF molecules on the surface of each cell type after addition of TAPI-1 were fitted to Equation 10 to estimate *k_synth_* for that cell type. Displayed data represent TNF synthesis by day 4 granuloma cells for three hours in the presence of TAPI-1. Error bars indicate standard deviations. Values of R^2^ for curve fitting for mDCs, macrophages and pDCs are 0.97, 0.99 and 0.98, respectively.

Using TAPI-1 as a TACE inhibitor to suppress the release of TNF from the membrane of TNF-expressing cells over a 3 hour time course, the rates of mTNF synthesis *k_synth_* by different types of immune cells in granulomas isolated at 2 and 4 days were measured by flow cytometry as described in Methods. TAPI-1 at a concentration of 100 µM was shown to partially suppress the TACE-mediated release of TNF from the cell membrane, so that the rate constant for TNF release after addition of TAPI-1 *k'_TACE_* was not zero. A higher concentration of TAPI-1 (200 µM) has been shown to have stronger inhibitory effects on the release of TNF from the membrane of human peripheral blood T cells [50], [66]. However, we found that high concentrations of TAPI-1 induce cell death. Thus, the rate of mTNF synthesis by each cell type was quantified by fitting experimental data to Equation 10 as described in Methods and shown in Figure 5. The results of the fit for *k_synth_*, *k_TACE_* and *k'_TACE_* from three experiments are averaged and reported in Table 8. Interestingly, PPD-bead granuloma T cells and B cells did not express quantifiable amounts of mTNF, although proinflammatory T cells have been reported to produce TNF in Mtb-infected mice [68]. To our knowledge, this is the first experimental quantification of the rate of TNF synthesis by granuloma-comprising immune cells.

**Table 8:** Average rate of mTNF synthesis and average rate constant for TNF release quantified by multi-color flow cytometry for different types of TNF-expressing immune cells (isolated from 10 mice) isolated from day 2 and 4 granulomas.

Cell type	*k_synth_* (#/cell.sec): day 2	*k_synth_* (#/cell.sec): day 4	*k_TACE_* (s^−1^)*	*k'_TACE_* (s^−1^)*
Lymphoid dendritic cells	1.01±0.74	0.81±0.35	(4.23±1.23)×10^−4^	(3.27±0.87)×10^−4^
Myeloid dendritic cells	0.26±0.21	0.21±0.05	(4.49±1.86)×10^−4^	(3.09±1.45)×10^−4^
Macrophage	0.17±0.09	0.15±0.03	(4.55±1.36)×10^−4^	(3.18±1.16)×10^−4^

*: Values of *k_TACE_* and *k'_TACE_* were averaged over all data on day 2 and day 4 granuloma cells.

### TNF/TNFR binding and trafficking dynamics and cellular organization control TNF availability within a granuloma

In general, the differences between experimental data on day 2 and 4 granulomas, although significant, are fairly small. Thus, using our data on cellular composition, cell-specific rates of mTNF synthesis and TNFR densities from day 4 PPD-bead granulomas as well as literature data on TNF/TNFR kinetic parameter estimates as inputs to our model, we studied mechanisms that control steady state TNF availability within a TB granuloma. Here we illustrate the role of two important factors, (i) molecular level processes governing TNF/TNFR interactions and intracellular dynamics and (ii) cellular organization within the granuloma, in regulating TNF availability within a granuloma.

**Figure 6:**
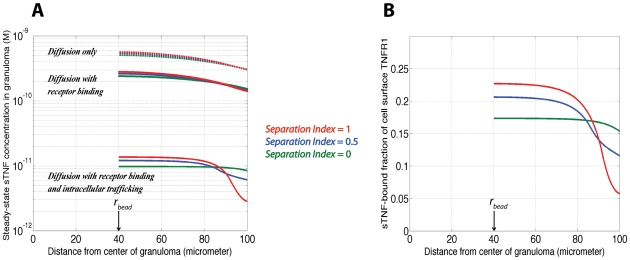
Predictions of the two-compartment model for a PPD bead granuloma. (A) The effects of receptor binding, intracellular trafficking of TNF and cellular organization within granuloma (represented by separation) on the steady state spatial distribution of free sTNF in a granuloma. (B) The effect of separation between different cell types in a granuloma on the spatial concentration of sTNF-bound cell surface TNFR1. Parameter values for the rate of mTNF synthesis (and similarly for TNFR densities) in each compartment were computed via Equations 6 and 7, using experimental data for day 4 granulomas presented in Figure 4 and Tables 7 and 8. Other parameter values are as listed in Table 3. The qualitative aspects of these plots are similar for day 2 granulomas.

To study the influence of TNF-associated molecular level processes on the availability of TNF, and thus TNF signaling within a TB granuloma, the distribution of sTNF in a granuloma was calculated by comparing modeling results in the presence of TNF intracellular trafficking with results of the model in the absence of TNF/TNFR internalization and shedding or TNF binding to TNF receptors. Figure 6A compares the spatial distributions of free sTNF at steady state for each case. TNF/TNFR reactions and interactions significantly affect the available amount of sTNF in a granuloma. Reversible binding of sTNF to cell surface receptors can reduce the amount of available extracellular sTNF in the granuloma by approximately two-fold. However, other molecular processes including the intracellular trafficking of TNF lead to a dramatic decrease of up to two orders of magnitude in the extracellular concentration of sTNF compared with the case in which TNF is produced and diffuses in a granuloma without binding to cell surface receptors. This result is consistent with experimental data on the role of TNFRs in modulating the biologic activity of TNF where a reduction of more than one order of magnitude in serum TNF levels of LPS-challenged control mice compared with TNFR-deficient mice has been observed [17].

Numerous studies have shown that TB granulomas, especially in humans as well as guinea pig and non-human primate models [9], [10], form as organized structures composed of a core of macrophages and DCs surrounded by a ring of lymphocytes. However, the effect of such a specific cellular organization on trafficking and availability of cytokines, in particular TNF, in the granuloma microenvironment has not been studied. To demonstrate the effect of spatial organization of immune cells on TNF availability in a granuloma, we performed simulations for varying levels of separation index (*s*) between populations of macrophages/DCs and lymphocytes within the granuloma (see Methods for more information). Our modeling results show that in the presence of TNF/TNFR binding and intracellular trafficking, the organization of cells within a granuloma significantly influences the availability of TNF. As such, greater levels of separation between macrophages/DCs and lymphocytes (separation index close to or at 1) result in steeper gradients of TNF concentration in the granuloma (Figure 6A). When the granuloma is organized in this way, the granuloma core (which is completely or almost completely composed of macrophages and DCs) is exposed to a higher concentration of TNF, while the mantle (which is composed of lymphocytes) is exposed to a lower concentration of TNF in comparison with the case of a zero separation index (reflecting a well-mixed cellular organization). A similar effect is observed for the number of sTNF-bound cell surface TNFR1 that controls the type and level of TNF-induced cell response in the granuloma (Figure 6B). For sufficiently large separation indices, a greater fraction of TNFR1 molecules on the membrane of macrophages and DCs in the granuloma core bind to sTNF in comparison with lymphocytes in the outer compartment. These results demonstrate that molecular level processes, including TNF intracellular trafficking and TNF receptor recycling, together with how immune cells (with different levels of TNF and TNF receptor expression) are organized within the granuloma control the amount of available TNF for each cell type and thus cell-specific TNF signaling.

### Effect of TNF-neutralizing drugs on availability of TNF within a granuloma

In order to study the effects of TNF-neutralizing drugs with various properties on the availability of TNF in a granuloma, we model a hypothetical TNF-neutralizing drug as an agent that diffuses from surrounding tissue into the granuloma, binds to TNF molecules and inhibits sTNF from binding to TNF receptors. We investigated how the efficiency of TNF neutralization (defined by Equation 9) by anti-TNF drugs is influenced by drug properties, including drug/TNF association and dissociation kinetics, drug ability to bind to mTNF, and drug/TNF binding stoichiometry. Three classes of hypothetical drugs with defined properties were modeled loosely based on properties of human TNF-neutralizing drugs (e.g. infliximab and etanercept) and their efficiencies of TNF neutralization were compared. Since the general behavior of all classes of drugs was shown to be independent of cellular organization in the granuloma (data not shown), model results for a separation index of one are discussed below.

### Class 1: drug binding to only sTNF at a binding ratio of 1∶1

**Figure 7:**
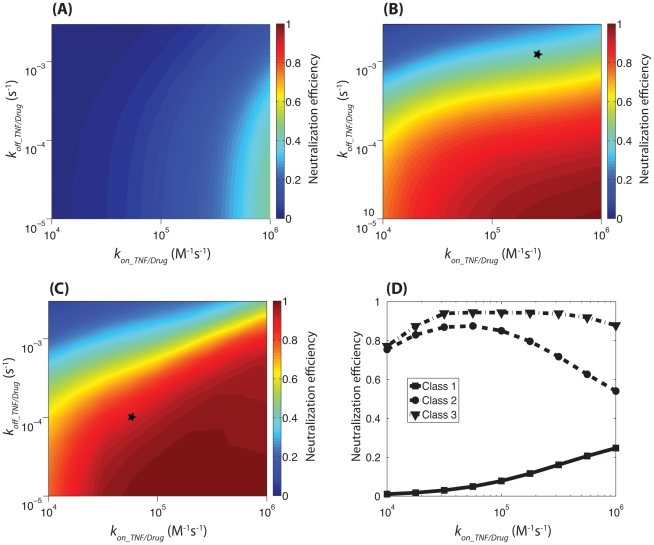
Model predictions for the effect of TNF-neutralizing drugs with various properties on the availability of TNF within a granuloma. (A) Class 1: the drug can only bind to sTNF with a binding ratio of 1∶1. (B) Class 2: the drug can bind to both mTNF and sTNF with a binding ratio of 1∶1. The star shows the location of a drug with TNF binding kinetics similar to etanercept. (C) Class 3: the drug can bind to both mTNF and sTNF with a binding ratio of 3∶1. The star shows the location of a drug with TNF binding kinetics similar to infliximab. (D) Model predictions for the effect of TNF/drug association rate constant on neutralization efficiency of drugs of different classes but identical affinities (*K_d_Drug_* = *k_off_TNF/Drug_*/*k_on_TNF/Drug_* = 10^−9^ M). Model parameter values are the same as Figure 6. TNF neutralization-associated parameter values are as listed in Table 4.

We first consider a drug that binds to sTNF with a binding ratio of 1∶1, inhibiting it from binding to both TNFR1 and TNFR2. The effects of varying association and dissociation rate constants (*k_on_sTNF/drug_* and *k_off_sTNF/drug_*) for sTNF and drug are shown in Figure 7A. Model results show that depending on sTNF/drug association and dissociation rate constants, 0%–50% of total available sTNF in a granuloma can be neutralized. As expected, drugs with greater affinities for sTNF more efficiently neutralize TNF in the granuloma. Interestingly, increasing *k_on_sTNF/drug_* without changing drug affinity leads to an increase in the drug neutralization efficiency (Figure 7D, Class 1). This is because drugs compete with cell surface TNFRs for binding to sTNF and thus a drug with a greater *k_on_sTNF/drug_* can neutralize larger amounts of sTNF.

### Class 2: drug binding to both sTNF and mTNF at a binding ratio of 1∶1

We next consider a drug that binds to both sTNF and mTNF with a binding ratio of 1∶1. We assumed identical association and dissociation rate constants for drug binding to mTNF and sTNF. TACE activity was considered independent of whether mTNF is bound to drug or not. Model results show that at all values of TNF/drug association and dissociation rate constants, a drug with the ability to bind to both sTNF and mTNF is more efficient in neutralizing TNF in a granuloma compared with a drug that can only bind to sTNF (compare Figures 7A and 7B). In other words, our model results demonstrate that even if sTNF is considered as the primary form of TNF that controls TNF-mediated signaling in granuloma cells, binding to mTNF is an important determinant of TNF neutralization power of the drug. This can be explained by rapid binding of diffusing drug molecules to mTNF in the absence of competition effects of cell surface TNFRs. However, similar to the case of Class 1 drug tested, TNF neutralization most efficiently occurs for a drug with the highest affinity for TNF.

Interestingly, among drugs with a constant affinity of *K_d_* = 10^−9^ M there is an optimum in neutralization efficiency that occurs for a drug with approximate values of *k_on_TNF/drug_* = 5.6×10^4^ M^−1^s^−1^ and *k_off_TNF/drug_* = 5.6×10^−5^ s^−1^ (Figure 7D, Class 2). To explain this result, we need to note that an mTNF/drug complex can be released into extracellular spaces due to TACE activity and then acts as a source for sTNF in the granuloma. When TNF/drug association is sufficiently rapid, drug binding to mTNF occurs before mTNF can be released into extracellular spaces. Thus, a significant proportion of sTNF in the granuloma is produced only after dissociation of sTNF from mTNF/drug complexes that are released from the cell membrane. In other words, the drug exerts a delay in the release of available sTNF from the cell membrane. Under these conditions, increasing TNF/drug dissociation rate constant increases the amount of sTNF dissociated from extracellular TNF/drug complexes and reduces the efficiency of TNF neutralization. This can explain why a drug of Class 2 type with intermediate values of TNF association and dissociation rate constants can more efficiently neutralize TNF compared with drugs of the same Class with the same affinity for TNF but higher values of these rate constants.

### Class 3: drug binding to both sTNF and mTNF at a binding ratio of 3∶1

Finally, we considered a drug that binds to both trimeric sTNF and mTNF molecules that possess three binding sites for the drug. An sTNF molecule with either one, two or three drug molecules bound is neutralized and not able to bind TNFR1 or TNFR2. This assumption helps us compare modeling results for TNF neutralization by different classes of drugs. Further, this assumption is consistent with experimental data indicating that only trimeric TNF is biologically active and that both monomeric TNF and artificially prepared dimeric TNF do not efficiently trigger TNF signaling in cells [69], [70]. We investigated the effect of multiple binding sites for drug binding to TNF and formation of larger drug/TNF complexes on the efficiency of TNF neutralization in a granuloma. Model results show that the drug concentration in the lung tissue is large enough that this greater drug/TNF binding ratio does not limit availability of the drug for binding to free TNF molecules. Thus, at large values of TNF/drug association rate constant, a higher binding ratio (i.e. 3∶1) increases the efficiency of TNF neutralization in comparison to a drug of Class 2 type with a binding ratio of 1∶1 (compare Figures 7B and 7C). However, binding stoichiometry does not significantly influence the level of TNF neutralization at low values of TNF/drug association rate constant, where TNFRs dominate the drug in competition for binding to sTNF (Figure 7C). An optimum in neutralization efficiency amongst Class 3 drugs of the same affinity *K_d_* = 10^−9^ M occurs in the same range of TNF/drug association and dissociation rate constants as observed for Class 2 (Figure 7D).

## Discussion

We have developed a two-compartment mathematical model that captures the structural features of a TB granuloma based on an experimental mouse PPD bead model and also includes molecular processes that govern the intracellular and extracellular trafficking of TNF. The model includes fine grain details at the level of TNF receptor dynamics, while using a coarse grain description for cellular level details representing a snapshot in time of a granuloma comprised of a static number of immune cells. This is based on a significant difference between the time-scale of TNF/TNFR associated molecular processes studied here and cellular level events that may change the structure of a granuloma (e.g. cell recruitment, migration and death).

The detailed consideration of synthesis, diffusion, receptor binding and intracellular trafficking of TNF within the heterogeneous three-dimensional structure of a granuloma distinguishes our model from a previous study by Marino *et al* on the role of TNF in host defense against TB [28]. The model developed by Marino *et al* describes the temporal dynamics of the immune response to Mtb infection in active and latent phases within a time course of 500 days by inclusion of TNF immunological functions on macrophages and T cells. However, we focus in this study on a snapshot in time of a granuloma to study the steady state spatial distribution of available TNF. We used results of our model sensitivity analysis as a novel tool to lead experiments to measure critical model parameters in artificial granulomas induced in the lungs of mice following injection of mycobacterial PPD-coated beads. Finally, whereas TNF neutralization has been simulated by Marino *et al* via removing fractions of available sTNF and/or mTNF, we studied the effects of TNF-neutralizing drugs by incorporation of their mTNF and/or sTNF binding kinetics and stoichiometry.

Model analysis helped us characterize two mechanisms for controlling the availability of TNF within a granuloma. These mechanisms include intracellular trafficking of TNF via internalization of recyclable TNFRs, and specific cellular organization within the granuloma, i.e. the level of separation between different classes of cells. Further, we demonstrated that for the resulting effect of cellular organization on spatial distribution of available TNF in the granuloma to be significant, intracellular trafficking of TNF is essential (Figure 6A). Hence, the spatial heterogeneity in the level of TNF and TNFR expression, and thus the amount of TNF internalization that occurs as a result of specific organization of different cell types in the granuloma controls the spatial distribution of the available amount of TNF for signaling for each specific cell type.

For sufficiently large values of the separation index in the granuloma, the model predicts significantly greater levels of sTNF binding to TNFR1 on the membrane of macrophages/DCs in the core compared with lymphocytes in the mantle (Figure 6B), which might be important for spatially coordinating the TNF-induced immunological functions for cells in a granuloma. Rangamani and Sirovich have recently shown via mathematical modeling that the induction of the two major TNF-induced signaling pathways, the caspase-mediated apoptotic pathway and the NF-κB-mediated survival pathway, are primarily controlled at the level of TNF/TNFR1 interactions [71]. As such, very low initial concentrations of TNF (i.e. less than 10^−11^ M) that can activate only a limited number of cell surface TNFR1 molecules are not capable of inducing apoptosis in the cells [52]. However, efficient NF-κB activation has been reported at TNF concentrations as low as 10^−13^ M based on both modeling [72] and experimental analysis of TNF signaling in HL60 cells and 3T3 mouse embryonic fibroblasts [73], [74]. Further, TNF/TNFR2 interactions have been shown to enhance TNFR1-dependent activation of caspase-mediated apoptotic pathway [75], [76]. These suggest a differential induction of apoptotic and survival signaling pathways between the granuloma core that is comprised of macrophages/DCs and the surrounding ring of lymphocytes.

**Figure 8:**
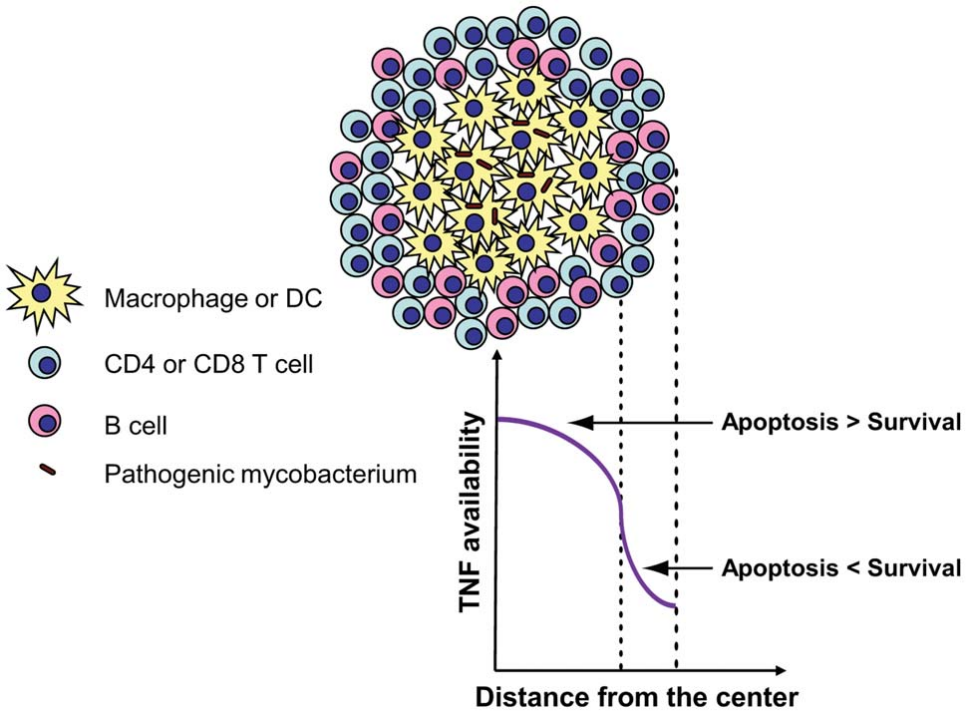
Spatial coordination of the TNF-induced immunological functions in a classical granuloma composed of a core of macrophages and DCs surrounded by a ring of lymphocytes. Great availability of TNF in the core of granuloma (together with TNF-induced TNFR2 activation) can turn on the TNFR1-dependent caspase-mediated apoptotic pathway that favors antigen cross-presentation as well as elimination of the pathogen inside the granuloma. Low level of TNF availability in the mantle of granuloma is sufficient to turn on the NF-κB signaling which favors cell survival and expression of pro-inflammatory genes but not the apoptotic pathway.

The hypothesis of differential induction of TNF-mediated signaling pathways for classical granulomas such as ones observed particularly in human, nonhuman primate and guinea pig models of TB [9], [10] has immunological implications. Whereas TNF-induced apoptosis of granuloma core macrophages that contain pathogenic mycobacteria is required for antigen cross-presentation and subsequent T cell priming and helps eliminate the pathogen [77], [78], lymphocyte (especially CD4 and CD8 T cell) death by TNF-induced apoptosis has been reported as one of the important components of an ineffective immune response against mycobacterial infections [79], [80]. However, the TNF-induced survival signaling pathway is required for retaining T cells at the developing granuloma site where they produce IFN-γ, activating macrophages in synergy with TNF to kill intracellular infections [81]. Thus, our novel hypothesis is that a separate cellular organization in the granuloma may favor an efficient immune response via spatially coordinating the TNF-induced immunological functions in the granuloma (Figure 8). Consistent with our hypothesis, very few apoptotic lymphocytes in classical TB granulomas induced in the guinea pig have been detected and most apoptotic cells have been seen close to the core of granulomas [82]. Further, because cellular organization undergoes dynamic changes with granuloma development and at different stages of immune response (innate versus adaptive) to TB infection, it can be a factor controlling the diverse activities of TNF according to the stage of infection in the lung tissue.

Finally, we used the model to predict and analyze the effects of TNF-neutralizing drugs with different properties on the availability of TNF within a developed granuloma. Average serum concentration of two murine analogs of human TNF blockers, infliximab and etanercept, after a single dose of drug, has been reported to be on the order of 10^−7^–10^−6^ M [64]. Using this reported concentration to estimate the tissue level concentration of these drugs (Table 4), we demonstrated that the efficiency of TNF neutralization within the granuloma not only depends on the affinity of the drug for TNF, but also on the ability of the drug to bind to mTNF versus sTNF, the rate constants for drug/TNF association and dissociation reactions as well as the drug/TNF binding stoichiometry (Figure 7).

We can use these modeling results to generate a hypothesis regarding experimentally observed differences in the neutralizing power of the two major human TNF blockers, infliximab and etanercept, based on their TNF binding specificities. Infliximab is a chimeric monoclonal TNF antibody that binds potently to both sTNF and mTNF. Up to three infliximab molecules can bind to one trimeric TNF molecule. Etanercept is a TNF receptor p75-IgG fusion protein that can also bind to both trimeric sTNF and mTNF. However, two receptor arms of etanercept contact two of the three receptor binding sites on different faces of the trimeric TNF, leading to a binding molar ratio of 1∶1 for TNF/etanercept binding. Further, etanercept has greater TNF association and dissociation rate constants in comparison with infliximab [48], [83], [84]. Hence, based on the classification of TNF-neutralizing drugs we presented in this paper, infliximab and etanercept can be considered as drugs of Class 3 and Class 2 types, respectively. Given the TNF binding stoichiometries and reported TNF association/dissociation rate constants for infliximab and etanercept (infliximab: *k_on_TNF/drug_* = 5.7×10^4^ M^−1^s^−1^, *k_off_TNF/drug_* = 1.1×10^−4^ s^−1^ and etanercept: *k_on_TNF/drug_* = 2.6×10^5^ M^−1^s^−1^, *k_off_TNF/drug_* = 1.3×10^−3^ s^−1^) [84], our granuloma model predicts TNF neutralization efficiencies of 0.90 and 0.39 for drugs with identical TNF binding properties to infliximab and etanercept, respectively; these efficiencies are marked with stars on Figures 7B, C. This is consistent with the reported higher TNF neutralization power of the TNF antibody (analog of infliximab) in comparison with the TNF receptor fusion molecule (analog of etenercept) in chronically Mtb-infected mice [85]. Although decreased penetration of the receptor fusion molecule into the lungs compared with antibody has been hypothesized to be a reason for the higher TNF neutralization power of antibody [85], we did not observe a significant change in the neutralization efficiency of simulated drug analogs by changing drug permeability in the granuloma (*k_c_*) by up to two order of magnitude (data not shown). Thus, the difference in binding properties of infliximab and etanercept must be considered when explaining the higher rate of TB reactivation induced from infliximab treatments in comparison with etanercept, although we anticipate that differential functional properties such as induction of apoptosis in TNF-producing cells by infliximab but not etanercept further influence the outcome of anti-TNF treatments [46], [86].

Although we have focused this study primarily on molecular and cellular scale processes within a snapshot of time in a granuloma, it will be necessary to consider multiple time and length scales (including dynamics in the lymphatic system) to further examine the role of TNF and anti-TNF therapies in the process of granuloma development and maintenance. We are currently working on this multi-scale approach [87].

## Supporting Information

Text S1 Simulation results for the spatial profiles of different forms of TNF in the model
(0.03 MB DOC)

Table S1 Parameters defined or modified based on incorporation of different cell types in the granuloma model
(0.07 MB DOC)

Table S2 Parameter sets used to generate curves on Figure 3
(0.04 MB DOC)

Figure S1 A schematic representation of parameters *s* and *f* used in the two-compartment model of PPD-bead granuloma. (A) Parameter *s* (separation index) is defined as indicated in Equation (5) to present the level of separation between different cell types in the granuloma model (other than sensitivity analysis) when all cell types are present. A separation index (*s*) of 0 is equivalent to a totally mixed cellular organization. Increasing *s* leads to an increase in the level of separation in the cellular organization as *s* = 1 represents a cellular organization in which macrophages and DCs are separate from but surrounded by lymphocytes. (B) Parameter *f* is defined as the fraction of cellular granuloma in the outer compartment and is only used when distinct cell types are not considered in the model (e.g. in sensitivity analysis). Increasing *f* results in a decrease in *r_core_* while *r_bead_* and *r_g_* are maintained constant.
(2.14 MB TIF)

Figure S2 Simulation results for the steady-state concentration profiles of the model species, including sTNF, sTNF/TNFR2_shed_, sTNF-bound and internalized TNFRs in a granuloma for two sets of parameter values: (A), (B) *k_synth_in_* = 1 #/cell.s, *k_synth_out_* = 0.01 #/cell.s, *R_1_in_* = *R_2_in_* = *R_1_out_* = *R_2_out_* = 2000 #/cell. (C), (D) *k_synth_in_* = *k_synth_out_* = 0.1 #/cell.s, *R_1_in_* = *R_2_in_* = *R_1_out_* = 500 #/cell , *R_2_out_* = 5000 #/cell. For both simulations, *s* = 1 and *f* = 0.5. Other parameter values are as listed in Table 3. Arrows indicate radius of the bead (*r_bead_*) and radius at which the two compartments are separated (*r_core_*).
(2.22 MB TIF)
