# Supplementary material for: Identification of Key Processes that Control Tumor Necrosis Factor Availability in a Tuberculosis Granuloma
Source: PLoS Comput Biol. 2010 May 6;6(5):e1000778. doi: 10.1371/journal.pcbi.1000778 (PMC2865521; doi:10.1371/journal.pcbi.1000778)
Supplement: Text S1 — Simulation results for the spatial profiles of different forms of TNF in the model (0.03 MB DOC) [file pcbi.1000778.s001.doc]

**Simulation results for the spatial profiles of different forms of TNF in the model**

Sample simulation results from the granuloma model for two sets of parameter values are presented in Supplementary Figure S2. This figure displays the spatial profiles of extracellular (free and shed TNFR2-bound) and cell-associated (membrane and internalized TNFR1- and TNFR2-bound) sTNF concentrations at steady state. The model predicts that the average free sTNF concentration in the granuloma (depending on parameter values) lies within the range of 10^-12^-10^-10^ M (~100-10,000 pg/ml). In the particular simulation indicated in Figure S2(A, B), TNF is mostly produced at the inner compartment of granuloma and diffuses outward (*ksynth_in > ksynth_out*), binding to TNF receptors with identical densities on all cells (*R1_in = R1_out* = *R2_in = R2_out*). For the set of parameter values considered here, the average sTNF-bound fraction of cell surface TNFR1 molecules in the inner and outer compartments are 0.66 and 0.07 and the average internalized fraction of TNFR1 molecules in the inner and outer compartments are 0.88 and 0.34, respectively. For a second simulation shown in Figure S2(C, D), TNF is homogeneously expressed in the whole granuloma (*ksynth_in = ksynth_out*), while binding to cell surface TNFR1 with the same compartment densities and TNFR2 with a higher density in the outer compartment (*R1_in = R1_out* = *R2_in < R2_out*). The average sTNF-bound fraction of cell surface TNFR1 molecules in the inner and outer compartments are 0.27 and 0.15 and the average internalized fraction of TNFR1 molecules in the inner and outer compartments are 0.76 and 0.62, respectively. These simulation results show that differential densities of TNFRs and the rate of TNF synthesis in granuloma compartments significantly influence the concentration profile of different forms of TNF in the granuloma.
