# Supplementary material for: Identification of Key Processes that Control Tumor Necrosis Factor Availability in a Tuberculosis Granuloma
Source: PLoS Comput Biol. 2010 May 6;6(5):e1000778. doi: 10.1371/journal.pcbi.1000778 (PMC2865521; doi:10.1371/journal.pcbi.1000778)
Supplement: Table S1 — Parameters defined or modified based on incorporation of different cell types in the granuloma model (0.07 MB DOC) [file pcbi.1000778.s002.doc]

**Table S1: Parameters defined or modified based on incorporation of different cell types in the granuloma model.**

| **Parameter** | **Parameter description** | **Value** |
| --- | --- | --- |
| *f_B_, f_CD4_, f_CD8_, f_Mac_, f_mDC_, f_pDC_* | Cell fractions of B cells, CD4 and CD8 T cells, macrophages, mDCs and pDCs in granuloma^*^ | Measured herein |
| *k_B_, k_CD4_, k_CD8_, k_Mac_, k_mDC_, k_pDC_* | Rate of mTNF synthesis by B cells, CD4 and CD8 T cells, macrophages, mDCs and pDCs in granuloma | Measured herein |
| *R1_B, R1_CD4, R1_CD8, R1_Mac, R1_mDC, R1_pDC* | TNFR1 density in B cells, CD4 and CD8 T cells, macrophages, mDCs and pDCs in granuloma | Measured herein |
| *R2_B, R2_CD4, R2_CD8, R2_Mac, R2_mDC, R2_pDC* | TNFR2 density in B cells, CD4 and CD8 T cells, macrophages, mDCs and pDCs in granuloma | Measured herein |
| *V_1_* (l) | Mean volume of a macrophage or dendritic cell^†^ | 1×10^-12^ |
| *V_2_* (l) | Mean volume of a lymphocyte^†^ | 3×10^-13^ |
| *l_g_* | Lymphocyte fraction in the whole granuloma | $f_{B}+f_{CD4}+f_{CD8}$ |
| *s* | Separation index | Varied from 0 to 1 |
| *l_o_* | Lymphocyte fraction in the outer compartment | $s(1-l_{g})+l_{g}$ |
| *f_1_* | Fraction of granuloma cells that compose macrophages and DCs in the inner compartment |  |
| *f_2_* | Fraction of granuloma cells that compose lymphocytes in the inner compartment |  |
| *f_3_* | Fraction of granuloma cells that compose macrophages and DCs in the outer compartment |  |
| *f_4_* | Fraction of granuloma cells that compose lymphocytes in the outer compartment |  |
| *V_in_* (l) | Mean cell volume in the inner compartment |  |
| *V_out_* (l) | Mean cell volume in the outer compartment |  |
| *ρ_in_* (cell/l) | Mean cell number density in the inner compartment |  |
| *ρ_out_* (cell/l) | Mean cell number density in the outer compartment |  |
| *r_core_* (μm) | Radius of the inner compartment | $[(r_{g}^{3}-r_{bead}^{3})\frac{f_{1}V_{1}+f_{2}V_{2}}{(1-l_{g})V_{1}+l_{g}V_{2}}+r_{bead}^{3}]^{\frac{1}{3}}$ |

**^*^** Cell fraction values measured in PPD bead granulomas were normalized before using in the mathematical model such that the sum of cell fractions was unity.

^†^ Average volumes of mouse macrophages and lymphocytes were calculated based on data from ^[1-3]^.

**References**

1. Morgan A, Talbot RJ. (1992) Effects of inhaled alpha-emitting actinides on mouse alveolar macrophages. Environ Health Perspect 97: 177-184.

2. Westermann J, Ehlers EM, Exton MS, Kaiser M, Bode U. (2001) Migration of naive, effector and memory T cells: Implications for the regulation of immune responses. Immunol Rev 184: 20-37.

3. Eurelings M, Notermans NC, Wokke JH, Bosboom WM, Van den Berg LH. (2002) Sural nerve T cells in demyelinating polyneuropathy associated with monoclonal gammopathy. Acta Neuropathol 103(2): 107-114.
