# Supplementary material for: Identification of Key Processes that Control Tumor Necrosis Factor Availability in a Tuberculosis Granuloma
Source: PLoS Comput Biol. 2010 May 6;6(5):e1000778. doi: 10.1371/journal.pcbi.1000778 (PMC2865521; doi:10.1371/journal.pcbi.1000778)
Supplement: Table S2 — Parameter sets used to generate curves on Figure 3 (0.04 MB DOC) [file pcbi.1000778.s003.doc]

**Table S2: Parameter sets used to generate curves on Figure 3.**

| **Simulation run** | **Parameter values^*^** |
| --- | --- |
| 1 | *ksynth_in* = 0.5, *ksynth_out* = 0.0, *R1_in* = 1000, *R2_in* = 1000, *R1_out* = 1000, *R2_out* = 1000, *f* = 0.4 |
| 2 | *ksynth_in* = 0.3, *ksynth_out* = 0.1, *R1_in* = 1000, *R2_in* = 1000, *R1_out* = 1000, *R2_out* = 1000, *f* = 0.4 |
| 3 | *ksynth_in* = 0.5, *ksynth_out* = 0.1, *R1_in* = 4000, *R2_in* = 1000, *R1_out* = 1000, *R2_out* = 1000, *f* = 0.6 |
| 4 | *ksynth_in* = 0.1, *ksynth_out* = 0.1, *R1_in* = 4000, *R2_in* = 1000, *R1_out* = 1000, *R2_out* = 1000, *f* = 0.4,  *k_int1_* = 5×10^-4^ |
| 5 | *ksynth_in* = 0.2, *ksynth_out* = 0.0, *R1_in* = 1000, *R2_in* = 1000, *R1_out* = 1000, *R2_out* = 1000, *f* = 0.7 |
| 6 | *ksynth_in* = 0.1, *ksynth_out* = 0.1, *R1_in* = 1000, *R2_in* = 1000, *R1_out* = 1000, *R2_out* = 1000, *f* = 0.4,  *K_d1_* = 1×10^-12^, *k_int1_* = 5×10^-4^ |
| 7 | *ksynth_in* = 0.1, *ksynth_out* = 0.1, *R1_in* = 1000, *R2_in* = 5000, *R1_out* = 500, *R2_out* = 1000, *f* = 0.4,  *K_d1_* = 1×10^-12^ |

^*^ Parameter definitions and their units are as defined in Table 3. Parameters unspecified in each run (including *D_1_*, *D_2_*, *φ*, *d_G_*, *ρ*, *r_g_*, *r_m_*, *r_bead_*, *r_core_*, *k_TACE_*, *k_deg_*, *K_d1_*, *K_d2_*, *k_on1_*, *k_on2_*, *k_int1_*, *k_int2_*, *k_shed_*, *k_rec1_*, *k_rec2_*, *k_t1_*, *k_t2_*, *k_deg1_*, and *k_deg2_*) have the same values as indicated in parentheses in Table 3.
