# Supplementary figures and images for: Identification of Key Processes that Control Tumor Necrosis Factor Availability in a Tuberculosis Granuloma
Source: PLoS Comput Biol. 2010 May 6;6(5):e1000778. doi: 10.1371/journal.pcbi.1000778 (PMC2865521; doi:10.1371/journal.pcbi.1000778)

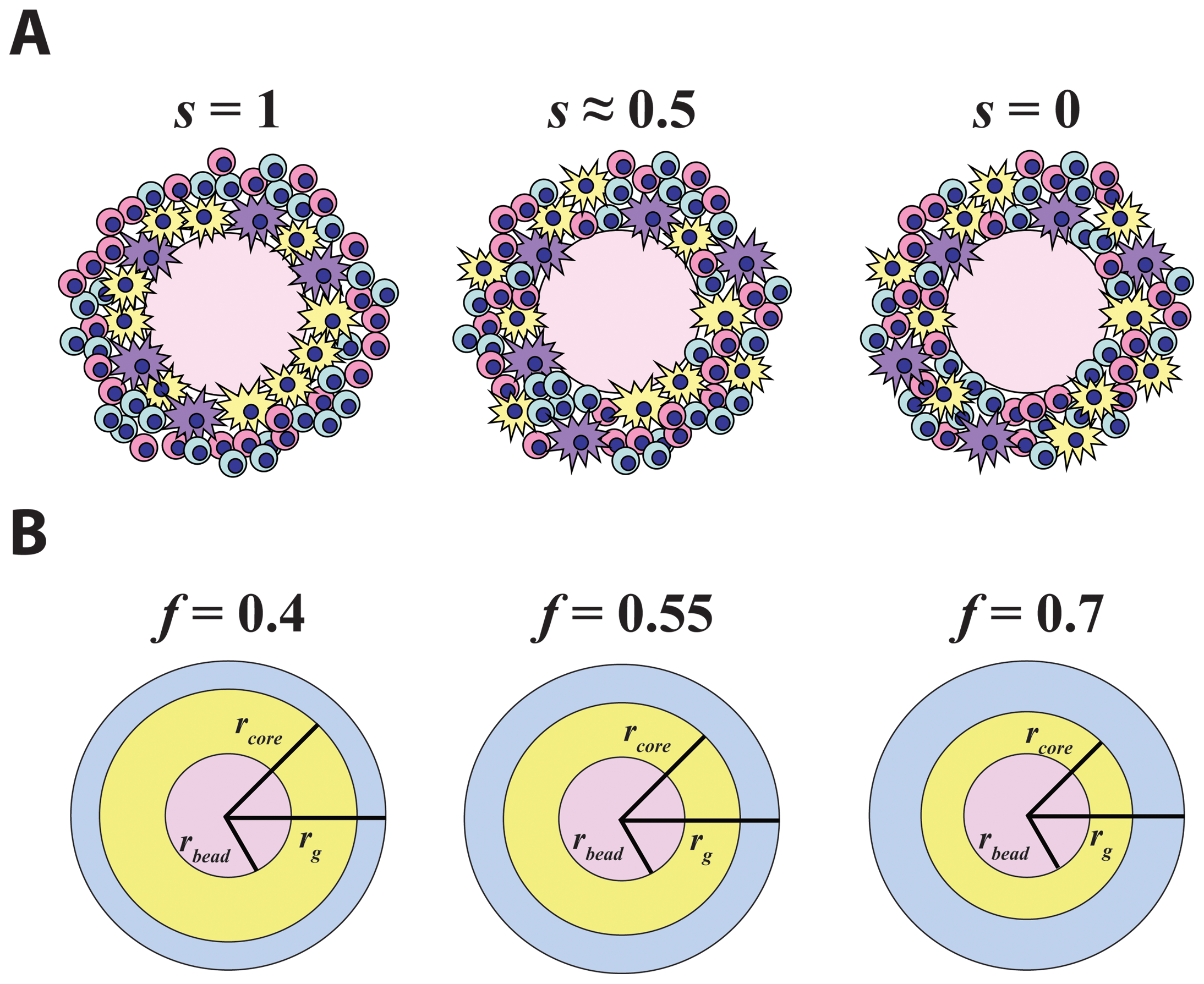

Supplement: Figure S1 — A schematic representation of parameters s and f used in the two-compartment model of PPD-bead granuloma. (A) Parameter s (separation index) is defined as indicated in Equation (5) to present the level of separation between different cell types in the granuloma model (other than sensitivity analysis) when all cell types are present. A separation index (s) of 0 is equivalent to a totally mixed cellular organization. Increasing s leads to an increase in the level of separation in the cellular organization as s = 1 represents a cellular organization in which macrophages and DCs are separate from but surrounded by lymphocytes. (B) Parameter f is defined as the fraction of cellular granuloma in the outer compartment and is only used when distinct cell types are not considered in the model (e.g. in sensitivity analysis). Increasing f results in a decrease in rcore while rbead and rg are maintained constant. (2.14 MB TIF) [file pcbi.1000778.s004.tif]

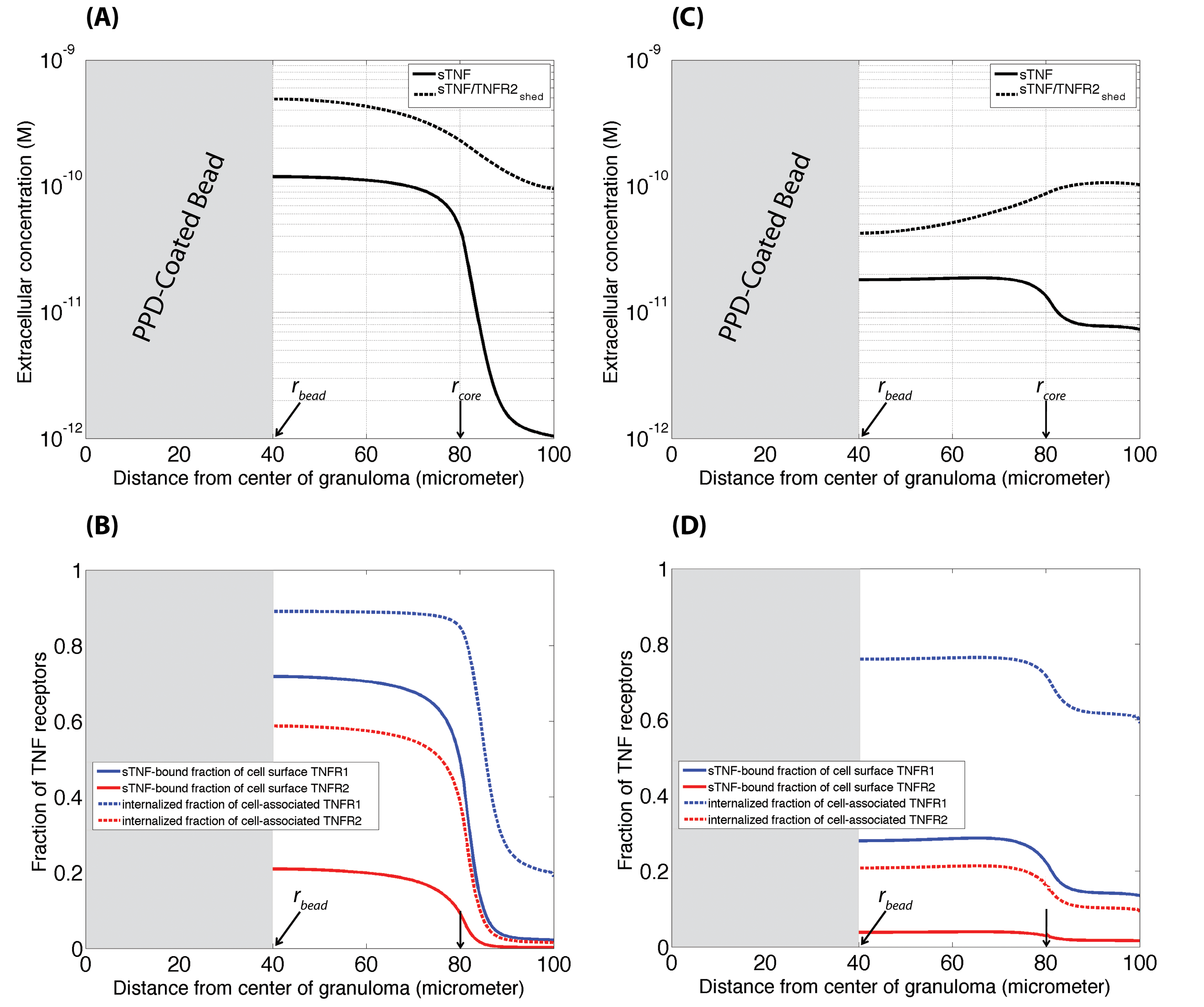

Supplement: Figure S2 — Simulation results for the steady-state concentration profiles of the model species, including sTNF, sTNF/TNFR2shed, sTNF-bound and internalized TNFRs in a granuloma for two sets of parameter values: (A), (B) ksynth_in = 1 #/cell.s, ksynth_out = 0.01 #/cell.s, R1_in = R2_in = R1_out = R2_out = 2000 #/cell. (C), (D) ksynth_in = ksynth_out = 0.1 #/cell.s, R1_in = R2_in = R1_out = 500 #/cell , R2_out = 5000 #/cell. For both simulations, s = 1 and f = 0.5. Other parameter values are as listed in Table 3. Arrows indicate radius of the bead (rbead) and radius at which the two compartments are separated (rcore). (2.22 MB TIF) [file pcbi.1000778.s005.tif]
